# The Impact of the Corona Virus Disease 2019 Pandemic on Chinese Middle School Students’ Self-Perceived Emotional States: A Wuhan Perspective

**DOI:** 10.3389/fpsyg.2021.740879

**Published:** 2021-11-05

**Authors:** Yan Huang, Jinyan Huang, Tingting Wang, Siying Xu, Jialing Li

**Affiliations:** ^1^Educational Economics and Management, Law and Business School, Wuhan Institute of Technology, Wuhan, China; ^2^The Research Institute for Educational Assessment and Research Methodology, School of Teacher Education, Jiangsu University, Zhenjiang, China; ^3^Logistics Management and Engineering, School of Management, Wuhan Textile University, Wuhan, China

**Keywords:** the COVID-19 pandemic, middle school students, emotional states, feelings of loss of control, negative emotions

## Abstract

This mixed-methods study examined the impact of the COVID-19 pandemic on 1493 Grades 7, 8, and 9 students’ self-perceived emotional states in Wuhan, China when it was locked down for the pandemic on January 23, 2020 and when the lockdown was lifted on April 8, 2020, as well as the changes of their emotional states over the 1-year period after the lockdown was lifted. A five-point Likert scale survey was administered to the participants between March 1 and April 1, 2020 when Wuhan was blocked down; and three focus group interviews were conducted between May 1 and May 31, 2021, 1 year after the lockdown was lifted. The results showed that these students in Wuhan experienced feelings of loss of control and negative emotions when the city was locked down and they were home quarantined; furthermore, there were significant differences for their self-perceived feelings of loss of control and negative emotions across demographic variables of gender, grade level, physical activity, social economic status, and family cohesion; finally, their emotional states changed substantially at different time nodes during this pandemic. Implications for students, parents, and schools are discussed.

## Introduction

The Corona Virus Disease 2019 (COVID-19) pandemic broke out suddenly at the beginning of 2020 in Wuhan, China and quickly spread across the whole country and other parts of the world (Chen et al., 2020, 2021; Dan, 2020; Li and Xu, 2020). Due to its highly contagious nature and unavailable treatment drugs, the public had to face a long period time of home quarantine, which not only increased the sensitivity of the general public to the new pandemic, but also posed a serious threat to people’s mental health (Dan, 2020; Liu, 2020; Liu et al., 2021).

In a public health crisis, risk perception plays an important role in affecting people’s mental health (Commodari et al., 2020; Ding et al., 2020). The public’s perceptions of risk as well as their emotional states are different; furthermore, the perceptions of cognitive risk are negatively correlated with depression in people in a public health crisis; therefore, when improving health policies for people’s mental health in public health crises, risk perception should be taken into consideration (Commodari et al., 2020; Ding et al., 2020).

Empirical studies have shown that the COVID-19 pandemic triggered emotional problems for the general public, such as anxiety and depression (Liu, 2020; Wang et al., 2020; Liu et al., 2021). Middle school students, in particular, are the most likely affected group by the COVID-19 pandemic (Commodari and La Rosa, 2020, 2021; Guessoum et al., 2020; Wang et al., 2021). They could not accurately identify the authenticity of information about this pandemic because of the rapid flood of complex information on the Internet and social media; moreover, they would have to study online at home because schools had been closed; consequently, they could easily develop feelings of loss of control and negative emotions; and the increased such feelings may lead to mental health problems such as anxiety and depression (Liu, 2020; Yu et al., 2020).

Since Wuhan first became the center of this pandemic, it is important to investigate the mental states of its middle school students during this major public health emergency when the entire city was locked down for the COVID-19 pandemic on January 23, during the lockdown of the city, and after the lockdown was lifted on April 8, 2020. Such investigations would provide important implications for the schools, middle school students, and their parents and guardians to cope with future major public health emergencies.

## A Summary of the Literature

Major public health emergencies refer to the sudden occurrence of major infectious diseases, mass diseases of unknown origin, major food and occupational poisoning, and other events that may cause serious damage to the public health (Liu Y. Y. et al., 2020). Research has shown that anxiety and depression are the most common mental health problems that the general public can easily develop under the major public health emergencies (Loh et al., 2005; Motreff et al., 2020; Og et al., 2020; Türk et al., 2021).

For example, the outbreak of the severe acute respiratory syndrome (SARS) in some areas of China at the end of 2002 posed a threat to the health and security of the public and many affected individuals experienced anxiety and depression (Leppin and Aro, 2009; Main et al., 2011). Similarly, after the COVID-19 pandemic outbreak at the beginning of 2020, the public experienced stress response, accompanied by such psychological disorders as anxiety and depression (Liu C. H. et al., 2020). Anxiety was the first to appear, depression was the next; the more individuals were in the worst-hit areas, the higher their anxiety and depression levels would be (Breslau et al., 2008; Liu C. H. et al., 2020).

Anxiety and depression are also common among middle school students (Su, 2006; Hankin et al., 2015; Ling, 2019). Adolescent anxiety and emotional disorders manifested as nervousness, crying, and irritability, accompanied by corresponding cognitive, behavioral changes, and physical symptoms (Su, 2006; Ling, 2019). The prevalence of anxiety has a long-term negative impact on different aspects of children and adolescents’ lives, and affects their cognitive, behavioral, and social functioning at different life stages, leading to education failure, low self-confidence, low self-esteem, and depression (Hankin et al., 2015; Ling, 2019).

Depression is a psychological or psychiatric term, indicating a series of symptoms such as decreased energy and feeling despair (Gong et al., 2019). Adolescent depression and mood disorders become a slow and long-term process, manifested by a sudden decline in academic performance, deterioration in friend relationships, reduction in social interaction or recreational activities, changes in diet, sleep disorders, frequent fatigue, feeling worthless, and hopeless (Su et al., 2011; Frison and Eggermont, 2015). Depressive symptoms including depressive emotions (i.e., feelings of sadness, unhappiness, or depression over an indefinite period of time) are commonly found in adolescents (Hankin et al., 2015). Before the end of puberty, approximately 20% of girls and 7% of boys experience such depressive symptoms (Angold et al., 2002).

Anxiety and depression are more common among adolescents (Seipp, 1991; Ferrari et al., 2013; Ingul and Nordahl, 2013; Chen et al., 2020; Commodari et al., 2020; Guessoum et al., 2020; Chen et al., 2021; Yang, 2021). Many research studies have shown that the prevalence of anxiety and depression is higher in women than in men (Madasu et al., 2019; Yang, 2021). For example, in 2018, the global prevalence of major depression for 12 months was 5.8% for women and 3.5% for men (Ferrari et al., 2013). Also, gender and age are significantly related to anxiety and depression symptoms (Madasu et al., 2019; Yang, 2021). Female adolescents exhibit a higher risk of anxiety and depression during the COVID-19 pandemic and older adolescents are more depressed than younger adolescents (Chen et al., 2020, 2021). Adolescents suffering from anxiety and depression may experience a variety of adverse consequences, such as learning difficulties, poor academic performance, dropping out, not adapting to social relationships, and even risk of suicide (Seipp, 1991; Ingul and Nordahl, 2013).

Moreover, family is one of the most important environments for the development of adolescents’ mental health (Tian and Li, 2005; Xie et al., 2008; Türk et al., 2021). The higher the degree of family dysfunction, the higher the levels of adolescents’ anxiety and depression would be (Xie et al., 2008). For example, adolescents with separated parents have higher levels of anxiety and depression (Türk et al., 2021).

Middle school students belong to the vulnerable groups (i.e., under the age of 18), and the outbreak of the COVID-19 pandemic has triggered them anxiety and depression to varying degrees (Chen et al., 2020; Lorenzo et al., 2021). Wang et al. (2020) conducted a study on the psychological status of school-age children and adolescents under the influence of the COVID-19 pandemic, and the results showed that 10.4 and 22% of the measured participants showed anxiety and depression symptoms, respectively, furthermore, the older adolescents were more likely to develop depression than the younger ones.

In addition, since the outbreak of the COVID-19 pandemic, middle school students have been learning online courses and carrying out daily activities indoors. This lifestyle change (e.g., long-term home isolation, reduced social activities, online lessons, and increased parent-child time) as well as the threat of infection may lead to anxiety and depression (Jiang et al., 2020). Without proper psychological interventions, anxiety and depression in adolescents tend to persist into adulthood, causing more serious effects (Wang et al., 2020).

According to recent research studies, factors such as physical activity, social economic status (SES), and family cohesion are found to influence the mental health of middle school students (White, 2000; Pickett et al., 2017; Biddle et al., 2018; Pavey and Brown, 2019; Guhn et al., 2020). The following is a literature review summary of these factors.

First, long-term outdoor and sports activities can improve middle school students’ mental health (Bélanger et al., 2019). Middle school students who regularly engage in physical activities are significantly less troubled by psychological stress than those with little physical activity (Ding et al., 1998; Biddle et al., 2018; Yan et al., 2019). This is because active physical activity can reinforce themselves and make them feel good, thereby helping them overcome obstacles and become active (Pickett et al., 2017). Long sitting time and less physical activity significantly increase the chance of developing depression symptoms (Pavey and Brown, 2019). However, physical activity can cultivate their positive self-awareness, provide a sense of belonging, improve physical function, and relieve emotional distress (Driver and Ede, 2009).

Furthermore, the low SES of the family is a recognized risk of poor mental health development in children (Reiss, 2013). It is directly or indirectly related to their emotional development and mental health (Amone-P’Olak et al., 2009). The common SES indicators are family per capita income, parents’ education level, and their occupational status (Amone-P’Olak et al., 2009). Studies have shown that when adolescents are exposed to stressful life situations, among the three indicators that reflect the family’s socioeconomic status, per capita income, parents’ education, and parents’ occupational status, adolescents with higher parents’ education levels are less likely to experience mental health problems, while family income per capita and parents’ occupational status have little predictability for adolescents’ mental health (Reiss et al., 2019). During the pandemic, prolonged family isolation, uncertainty about the future, and financial difficulties experienced by the family may all trigger personal psychological symptoms (Karaman et al., 2021).

Finally, family cohesion refers to the degree of mutual commitment, help, and support between family members (Goodyer, 1998; Zhao et al., 2008; Williams, 2013; Yoon and Lian, 2020). Whether the emotional relationship between family members is concordant and harmonious has an important impact on the mental health of middle school students (Williams, 2013). Bad family relationships are one of the important factors that may cause them psychological problems (Goodyer, 1998; Yoon and Lian, 2020). Their overall level of anxiety is significantly negatively correlated with family cohesion; the lower the family cohesion, the easier it is for children to show depression and anxiety disorders (Yang, 2001; Craig et al., 2021). This may be due to the lack of emotional communication and mutual support between parents and children, and the family atmosphere is not harmonious, which not only makes children and adolescents feel lonely, depressed, withdrawn and silent, but also makes them feel greater pressure and cause anxiety (Wu et al., 2005). Furthermore, when they find that they are connected with their family and can rely on the family in a difficult situation, the family has an important influence on their behaviors and attitudes, and they perceive that the higher the support from the family, the less likely they are to engage in negative behavior (Jhang, 2017). This kind of support and communication between family members may be an important factor in relieving stress (Marta, 1997; Li and Xu, 2020; Zhu et al., 2021).

Recently, researchers have started to examine the impact of the COVID-19 pandemic on people’s lives in different countries (Commodari and La Rosa, 2020; Commodari et al., 2020; Ding et al., 2020; Guessoum et al., 2020). For example, Ding et al. (2020) investigated the psychological wellbeing and behavioral responses of adults in China during the COVID-19 pandemic and reported that risk perception and its associated factors significantly affect the mental health of people in public health crises. Furthermore, Commodari and La Rosa (2020) investigated 978 (males = 339; females = 639) secondary school students’ psychological experience of quarantine during the COVID-19 pandemic in Italy. The results indicated that females showed more significant psychological negative feelings about the quarantine experience. Similarly, Commodari and La Rosa (2021) examined 1017 secondary school students’ distance learning experiences during the first wave of the COVID-19 pandemic in Italy. The results indicated that distance learning was associated with a significant increase in student workload and consequent psychological distress related to homework.

To sum up, previous research studies on the COVID-19 pandemic were cross-sectional in nature (e.g., Chen et al., 2020, 2021). Few of them used longitudinal data for analysis. Moreover, most of previous research studies used quantitative methods in their investigations. The use of qualitative data would provide more in-depth information about the topic being investigated. To bridge these gaps, this study used both quantitative (i.e., a five-point Likert scale survey measuring participants’ self-perceived feelings of loss of control and negative emotions) and qualitative (i.e., focus group interviews examining the changes of participants’ emotional states) research methods in its design. Furthermore, it combined cross-sectional data with longitudinal data in its analysis.

## Research Questions

This study aimed to examine the emotional states of middle school students in Wuhan City, when it was locked down for the COVID-19 pandemic on January 23, 2020, during the lockdown of the city, and after the lockdown was lifted on April 8, 2020. Specifically, the following four research questions guided the study: (a) what were the effects of gender, grade level, physical activity, SES, and family cohesion on participants’ emotional states as measured by their self-perceived feelings of loss of control and negative emotions? (b) What were their emotional states when Wuhan was locked down for the COVID-19 pandemic? (c) What were their emotional states when the lockdown was lifted in Wuhan? And (d) how did their emotional states change over the 1-year period after the lockdown was lifted?

## Materials and Methods

### Instruments

A five-point Likert scale survey and follow-up focus group interviews 1 year after were used for the data collection of this study. The survey consisted of a demographic information section and 20 items which required the participants to indicate their responses on a five-point Likert scale ranging from 1 (strongly disagree) to 5 (strongly agree).

The demographic information included participants’ gender (i.e., male and female), grade level (i.e., Grades 7, 8, and 9), physical activity (i.e., low, medium, and high), SES (i.e., low, medium, and high), and family cohesion (i.e., low and high) information. The physical activity variable was measured by the length of time for daily physical exercise and physical labor (e.g., cleaning the floor and washing the dishes); with three levels of low (less than 1 h), medium (1–2 h), and high (more than 2 h) physical activity. The SES variable was measured by the monthly home income; with three categories of low (less than RMB5000), medium (RMB5000–10,000), and high (more than RMB10,000) SES. The family cohesion variable referred to the degree of mutual commitment, help and support between family members; with two levels of low and high family cohesion.

These 20 five-point Likert scale items were selected from previous literature (Cohen et al., 1983; Zigmond and Snaith, 1983; Moos and Moos, 1994) and were used to measure participants’ self-perceived feelings of loss of control and negative emotions. Among the 20 items, 10 were reverse scored items (see Table 1). Participants’ self-perceived feelings of loss of control and negative emotions were calculated by summating and building averages of these 20 items. Average scores around 4 and above may indicate high level feelings of loss of control and negative emotions. The increased such feelings and negative emotions would indicate that participants were experiencing emotional swings, which might lead to mental health problems eventually.

**TABLE 1:** A description of the 20 items with factor loadings.

Item #	Brief description	*Factor 1	*Factor 2
2	I am unable to control the important things in my life.	0.34	
4	I feel upset because something unexpected is happening.	0.60	
6**	I often feel that things are going my way.	0.74	
8**	I am able to control irritations in my life.	0.79	
10**	I feel that I can control everything that is happening.	0.76	
12	I feel that I have many difficulties that I cannot overcome.	0.65	
14**	I feel that I am able to do things that I have to do.	0.69	
16	I feel angry because something out of my control is happening.	0.57	
18**	I feel confident about my ability to deal with problems.	0.71	
20	I feel stressed about things that I have to do.	0.63	
1	I feel worried about things I have to deal with.		0.67
3	I feel anxious about things I have to do.		0.57
5**	I laugh and feel relaxed.		0.61
7**	I am happy about things.		0.76
9	I have sudden feelings of panic.		0.67
11	I feel restless and cannot keep still.		0.73
13**	I enjoy reading books or watching television.		0.67
15	I feel like something frightening is going to happen.		0.74
17**	I look forward to happy things.		0.71
19**	I like things that I liked before.		0.73

*Factor 1 = self-perceived feelings of loss of control; Factor 2 = self-perceived negative emotions; **indicates reverse scored items.

The follow-up focus group interviews with selected participants consisted of six interview questions (see Table 2) that required them to describe their emotional states when Wuhan was locked down and when the lockdown was lifted as well as the changes of their emotional states over the 1-year period after the lockdown was lifted. The criteria for selecting the participants were (a) they must come from different schools at different districts and represent different grades; (b) they must have gone through the entire process of Wuhan’s lockdown and its lifted lockdown; and (c) they must come from different SES and gender backgrounds. The researchers believed that the selected participants are representative of the main sample.

**TABLE 2:** Main themes of focus group interviews for research questions #2, #3, and #4.

Research questions	Interview questions	Main themes		
		Grade 7 group	Grade 8 group	Grade 9 group
#2: What were participants’ emotional states when Wuhan was locked down for the COVID-19 pandemic?	a) What was your feeling when you learned that Wuhan was locked down? b) During the home isolation period, how were you feeling?	a) Surprised b) Worried c) Panic d) Happy	a) Terrified b) Fearful c) Scared d) Bored	a) Terrified b) Fearful c) Scared d) Depressed
#3: What were participants’ emotional states when the lockdown was lifted?	a) What was your feeling when you learned that the lockdown was lifted? b) At the end of the home isolation period, how were you feeling?	a) Cheerful b) Worried c) Sad	a) Cheerful b) Anxious c) Calm	a) Excited b) Worried c) Relaxed
#4: How did participants’ emotional states change over the past 1 year?	a) What was your feeling today, 1 year after the lockdown was lifted? b) What were the changes in your emotional states from the lockdown to the lifted lockdown, and then to today?	Surprised- happy- worried	Fearful- cheerful- relaxed	Terrified- excited- proud

### Participants

The researchers first invited all the Grades 7–9 students studying at 22 middle schools across five districts of Wuhan City to participate in this study by providing them and their parents with letters of information and consent forms. With their parents’ permissions a total of 2000 students finally agreed to participate in the study. The total number of students in these 22 schools was 32,200 (see Appendix Table 1). These students were considered a representative sample of all middle school students in Wuhan. They were grouped by gender, grade level, physical activity, SES, and family cohesion for data analysis.

### Data Collection Procedures

Data were collected at two phases. The first phase was survey data collection, which was completed from March 1 to April 1, 2020 when Wuhan was locked down for the COVID-19 pandemic. Due to the limited availability and accessibility of participants, the second phase was focus group interview data collection with 15 middle school students (three groups representing Grades 7, 8, and 9) who had participated in the first phase of the study; the focus group interviews were conducted 1 year after the lockdown was lifted (from May 1 to 31, 2021) in Wuhan. Survey data collection was conducted online with the assistance of head teachers or mental health teachers at each middle school. Focus group interviews were also conducted online between the researchers and selected participants. Although research ethics committees have not been established in Chinese colleges and universities and they do not mandate ethics reviews for non-medical research involving human participants, the researchers provided all the participants and their parents with letters of information and consent forms; and they all understood that the participation was totally voluntary and their responses were strictly confidential.

The researchers sent out the link of the survey electronically for the students who had agreed to participate in the study to fill out online anonymously in the classroom with the assistance of their teachers. The survey questions were not mandatory. They could choose not to answer any questions that they felt uncomfortable with. During this data collection process, 507 (25%) students chose not to complete the survey. A total of 1493 participants submitted their completed surveys, with a response rate of 75%. There were no missing values in the received 1493 surveys.

### Data Analysis

Using SPSS, survey data were analyzed at different levels. First, exploratory factor analysis (EFA) was conducted to examine the construct validity of the survey. Second, after the correct number of factors was identified, the reliability (i.e., internal consistency) of the survey was calculated. Third, descriptive statistics, one-factor MANOVAs for demographic variables of gender, grade, physical activity, SES, and family cohesion were performed to examine significant group differences. It is important to note that since there are relatively small differences among these 22 middle schools in terms of their sizes and academic programs (see Appendix Table 1), significant differences among schools were not expected; and therefore, demographic variable of school was not included in the quantitative data analysis.

Furthermore, as previously mentioned, the 20 five-point Likert scale items were used to measure participants’ self-perceived feelings of loss of control and negative emotions. They were calculated by summating and building averages of these 20 items. Because there was likely to be content overlap or multicollinearity among the summated and averaged scores, separate one-factor MANOVA analyses were employed to test for significant between-group differences (Casado and Dereshiwsky, 2001).

The qualitative data were first coded and sorted, then organized, and finally grouped and categorized according to the recurring themes (Creswell, 2014). It is important to note that all the researchers have rich qualitative data analysis experience. They first aligned the focus group interview questions with the corresponding research question, and then entered qualitative data into Excel spreadsheets. Specifically, the procedures for analyzing qualitative data included (a) finding codes; (b) connecting codes; (c) sorting codes into different categories and subcategories individually, (d) organizing categories and subcategories collaboratively by content, (e) discussing conceptually similar responses, (f) grouping them together, and (g) categorizing them by the recurring themes. This process was to ensure inter-coder reliability of the qualitative data analysis. Also, to enhance the validity, direct quotes from the participants were incorporated (Creswell, 2014).

## Results

### Demographic Characteristics of the Participants

A total of 1493 middle school students became the participants of this study. Among them, 778 (52.1%) were male and 715 (47.9%) were female; 489 (32.8%), 489 (32.8%), and 515 (34.5%) were Grades 7, 8, and 9 students, respectively, 652 (43.7%), 486 (32.6%), and 355 (23.8%) reported low, medium, and high levels of physical activity, respectively, 500 (33.5%), 607 (40.7%), and 386 (25.9%) came from low, medium, and high SES families, respectively, 789 (52.8%) were from low cohesion families and 704 (47.2%) high cohesion families. The participants of this study were fairly balanced across the demographic variables of gender, grade, physical activity, SES, and family cohesion. Furthermore, 15 middle school students representing three middle schools were selected to participate in the focus group interviews. There were three focus groups with five participants in each group, representing Grades 7 (one male and four female students), 8 (all five female students), and 9 (three male and two female students), respectively.

### The Construct Validity and Internal Consistency Reliability of the Instrument

A maximum likelihood with promax rotation EFA was conducted to examine the construct validity of the 20 items. The Kaiser-Meyer-Olkin (KMO) measure of sampling adequacy was 0.85. Eigenvalues for two factors were > 1; furthermore, the scree plot suggested a two-factor model which explained 52% of the total variance (see Table 1). All items had moderate to high loadings (>0.30) on the two common factors.

Specifically, ten items (i.e., items #2, 4, 6, 8, 10, 12, 14, 16, 18, and 20) had moderate to high loadings on the first common factor; ten items (i.e., items #1, 3, 5, 7, 9, 11, 13, 15, 17, and 19) had moderate to high loadings on the second common factor. As shown in Table 1, the first factor addresses their self-perceived feelings of loss of control, and the second factor indicates their self-perceived negative emotions.

The instrument has been shown to be reliable, with alpha reliability coefficients of 0.92; furthermore, the 10 items for self-perceived feelings of loss of control as well as the items for self-perceived negative emotions have also been shown to be reliable, with alpha reliability coefficients of 0.89 and 0.90, respectively.

### Findings of the Quantitative Analysis

The one-factor MANOVAs for the each of the demographic variables of gender, grade, physical activity, SES, and family cohesion and major demographic variables were conducted to answer the research question #1, i.e., what were the effects of gender, grade level, physical activity, SES, and family cohesion on participants’ emotional states as measured by their self-perceived feelings of loss of control and negative emotions? These MANOVAs yielded significant between group differences, respectively. The results are presented in Table 3.

**TABLE 3:** MANOVA results comparing status differences.

**Multivariate Wilks’ Lambda tests**										

**Demographic variable**		**Value**		***F***		**Sig**				**Effect size**

Gender		0.946		42.258		**				0.054
Grade		0.972		10.703		**				0.014
Physical activity (PA)		0.658		173.582		**				0.189
SES		0.802		86.932		**				0.105
Family cohesion (FC)		0.882		100.081		**				0.118

**Tests of between-subjects effects**										

**Variable**		**Group mean**						**Sig.**		**Effect size**

Feelings of loss of control	Male (3.72)		Female (4.32)					**		0.036
Negative emotions	Male (3.63)		Female (4.03)					**		0.047
Feelings of loss of control	Grade 7 (3.83)		Grade 8 (3.89)		Grade 9 (4.28)			**		0.016
Negative emotions	Grade 7 (3.69)		Grade 8 (3.74)		Grade 9 (4.03)			**		0.027
Feelings of loss of control	Low PA (4.86)		Medium PA (3.76)		High PA (2.78)			**		0.274
Negative emotions	Low PA (4.26)		Medium PA (3.78)		High PA (3.08)			**		0.257
Feelings of loss of control	Low SES (4.89)		Medium SES (3.79)		High SES (3.19)			**		0.180
Negative emotions	Low SES (4.23)		Medium SES (3.75)		High SES (3.41)			**		0.122
Feelings of loss of control	Low FC (4.46)		High FC (3.49)					**		0.093
Negative emotions	Low FC (4.08)		High FC (3.53)					**		0.092

**Significant at p < 0.01.

*PA, physical activity; SES, socioeconomic status; FC, family cohesion.*

As shown in Table 3, the most commonly reported multivariate Wilks’ Lambda tests for demographic variables gender [*Lambda* (2.1490) = 42.26, *p* < 0.01, effect size = 0.054], grade [*Lambda* (4.2978) = 10.70, *p* < 0.01, effect size = 0.014], physical activity level [*Lambda* (4.2978) = 173.58, *p* < 0.01, effect size = 0.189], SES [*Lambda* (4.2978) = 86.93, *p* < 0.01, effect size = 0.105], and family cohesion [*Lambda* (2.1490) = 100.08, *p* < 0.01, effect size = 0.118] yielded significant findings. Also the significant *p*-value (<0.01) indicated individual between-group differences for both dependent variables of self-perceived feelings of loss of control and negative emotions.

Follow-up univariate ANOVAs indicated that there were significant differences for both dependent variables of self-perceived feelings of loss of control and negative emotions across all five demographic variables. Specifically, the female students experienced significantly higher level feelings of loss of control [*F*(1, 1491) = 55.55, *p* < 0.01, effect size = 0.036] and negative emotions [*F*(1, 1491) = 73.55, *p* < 0.01, effect size = 0.047] than the male students; Grade 9 students experienced significantly higher level feelings of loss of control [*F*(2, 1490) = 11.81, *p* < 0.01, effect size = 0.016] and negative emotions [*F*(2, 1490) = 20.30, *p* < 0.01, effect size = 0.027] than Grades 7 and 8 students; however, there were no significant differences between Grade 7 and Grade 8 students in both self-perceived feelings of loss of control and negative emotions.

Moreover, there were significant differences among students with low, medium, and high physical activities in terms of their self-perceived feelings of loss of control [*F*(2, 1490) = 281.57, *p* < 0.01, effect size = 0.274] and negative emotions [*F*(2, 1490) = 257.71, *p* < 0.01, effect size = 0.257]. Students with low physical activities experienced significantly higher level feelings of loss of control and negative emotions than students with medium and high physical activities (*p* < 0.01); also, students with medium physical activities experienced significantly higher level feelings of loss of control and negative emotions than students with high physical activities (*p* < 0.01).

Similarly, there were significant differences among students with low, medium, and high SES in terms of their self-perceived feelings of loss of control [*F*(2, 1490) = 163.32, *p* < 0.01, effect size = 0.180] and negative emotions [*F*(2, 1490) = 103.64, *p* < 0.01, effect size = 0.122]. Students with low SES experienced significantly higher level feelings of loss of control and negative emotions than students with medium and high SES (*p* < 0.01); also, students with medium SES experienced significantly higher level feelings of loss of control and negative emotions than students with high SES (*p* < 0.01).

Finally, the follow-up univariate ANOVAs indicated that students from low cohesion families experienced significantly higher level feelings of loss of control [*F*(1, 1491) = 152.48, *p* < 0.01, effect size = 0.093] and negative emotions [*F*(1, 1491) = 151.62, *p* < 0.01, effect size = 0.092] than students from high cohesion families.

### Findings of the Qualitative Analysis

Three focus group interviews with 15 middle school students were conducted to answer research questions #2, #3, and #4, respectively, about the participants’ emotional states when Wuhan was locked down for the COVID-19 pandemic on January 23, 2020 and when the lockdown was lifted on April 8, 2020 as well as the changes of their emotional states over the 1-year period after the lockdown was lifted. Table 2 presents a summary of the main themes of the three focus group interviews.

#### Participants’ Emotional States When Wuhan Was Locked Down for the Corona Virus Disease 2019 Pandemic

As shown in Table 2, Grade 7 participants reported that they were feeling surprised, worried, panic, and even happy when Wuhan was locked down for the COVID-19 pandemic. Grade 8 participants described that they felt terrified, fearful, scared, and bored when they heard about the lockdown of the city. Similarly, Grade 9 participants were feeling terrified, fearful, scared, and depressed at the time when Wuhan was locked down.

Four out of five Grade 7 participants described that they were feeling surprised, worried, and panic when Wuhan was locked down for the COVID-19 pandemic. “*On hearing the news, I was surprised*,” “*when hearing the news about the lockdown of Wuhan city, my first feeling was panic and worried*,” and “*I worried that the virus would spread to myself and my family*” were their common feelings. However, one male participant indicated that he was feeling happy because his parents went out to work every day and rarely spent time with him at home; but after the lockdown of the city he could stay with them every day and enjoyed the time together.

Grades 8 participants felt terrified, fearful, scared, and bored on hearing the news that Wuhan was locked down for the COVID-19 pandemic. “…*I could hardly believe that the virus was so infectious*,” “*my mother is a community worker and she may be affected*,” and “*my father works in another city, and I am scared that he may get the virus*” were their common feelings. Unlike her peers, one participant mentioned that she felt bored at home all day and night.

Similarly, Grades 9 participants felt terrified, fearful, scared, and depressed when they heard the news that Wuhan was locked down. One female participant mentioned that she had visited crowded places a few days before the lockdown of the city and was terrified that she might have been affected. Other participants made the following comments: “*when I heard the news, I was scared*,” and “*when I learned that Wuhan was the most seriously affected city, I was scared to death*.” Several participants emphasized that the atmosphere at their homes became depressing when they saw the increasing death toll on the news.

#### Participants’ Emotional States When the Lockdown Was Lifted

As shown in Table 2, Grade 7 participants reported that they were feeling cheerful, worried, and even sad when the lockdown was lifted. Grade 8 participants described that they felt cheerful, anxious, and calm when they heard that the lockdown of the city was lifted. Similarly, Grade 9 participants were feeling excited, worried, and relaxed at the time when the lockdown was lifted in Wuhan.

Four out of five Grade 7 participants described that they were feeling cheerful and anxious when the lockdown was lifted in Wuhan. “*I was very cheerful because I should be able to see my classmates and friends again*,” “*I could finally go outside to breathe fresh air*,” and “*I am worried about the recurrence of the pandemic*” were their common feelings. However, the one male participant indicated that he was feeling sad because his parents would go out to work every day and hardly spend time with him at home.

Grades 8 participants felt cheerful, anxious, and calm on hearing the news that the lockdown was lifted in Wuhan. “*I felt very cheerful because I could go wherever I want*,” “*I was very happy because I could meet my classmates and have dinner with them in our favorite restaurant*,” and “*I thought the virus would not go away easily and I felt anxious about it*” were their common feelings. Unlike her peers, one participant mentioned that she did not feel very excited but pretty calm because she had been used to staying at home with her family members.

Similarly, Grades 9 participants felt excited, worried, and relaxed when they learned that the lockdown was lifted. One female participant commented that she felt very happy because her family and friends were safe and sound. Other participants made the following comments: “*I was so excited and could not believe that the pandemic had been brought under control*,” and “*I felt worried that the virus might come back again.*” Several participants emphasized that the atmosphere at their homes became relaxing when they saw the new case was zero on the news.

#### Changes of Participants’ Emotional States Over the Past Year

As shown in Table 2, Grade 7 participants reported that their emotional states changed from being surprised to happy and then to worried from the lockdown to the lifted lockdown, and then to today. Grade 8 participants’ emotional states changed from being fearful to cheerful and then to relaxed. Slightly differently, Grade 9 participants’ emotional states changed from being terrified to excited and then to proud at these different time nodes.

Grade 7 participants described the changes of their emotional states at the three time nodes as being surprised-happy-worried. For example, one female participant provided the following description, “*I was first surprised to learn that Wuhan was locked down due to the COVID-19 pandemic and then felt happy when the lockdown was lifted in April 2020, but felt worried today because the COVID-19 pandemic was still a public health threat worldwide*.” Several participants added that the pandemic had become a serious threat to middle school students’ mental health.

Grade 8 participants felt fearful first, and then cheerful, and now relaxed. These changes of their emotional states were clearly reflected in one female participant’s description, “*I was fearful that the virus would spread to my family when the city was locked down; I felt happy and cheerful when the lockdown was lifted because the COVID-19 pandemic was under control; and now I feel relaxed because my life has become normal again*.”

Similarly, Grade 9 participants felt terrified first, and then excited, and now proud. One male participant’s comments clearly indicted these changes: “*I was very terrified when the city was locked down for the pandemic, and I did not know how to deal with it; afterwards, I felt excited when the lockdown was lifted. China is now the safest country in the world and I become very proud of being a Chinese citizen*.”

To sum up, the participants in the three focus groups experienced similar feelings at different time nodes. Most of them were surprised and scared when they heard the news of the lockdown of Wuhan City. They became cheerful and excited when the lockdown was lifted. One year after the lockdown was lifted, students returned to their schools and their life became normal. It is interesting to mention that Grade 9 students expressed their trust and pride in their home country.

## Discussion and Conclusion

The first research question was about the effects of gender, grade level, physical activity, SES, and family cohesion on Wuhan middle school students’ emotional states as measured by their self-perceived feelings of loss of control and negative emotions. Significant differences were found across all five demographic variables. Female students, Grade 9 students, students with low physical activities and SES, and students from low cohesion families experienced significantly higher level feelings of loss of control and negative emotions than male students, Grades 7 and 8 students, students with medium and high physical activities and SES, and students from high cohesion families, respectively. These results were consistent with the previous research findings (Ding et al., 1998; Reiss, 2013; Pavey and Brown, 2019; Yan et al., 2019; Chen et al., 2020; Commodari and La Rosa, 2020; Wang et al., 2020; Yoon and Lian, 2020).

It is important to note that the obtained effect size was small for each of these MANOVAs, which yielded significant group differences. Effect size identifies the strength of the conclusions about groups; and it often provides a more practical reading of the results (Creswell, 2014). The obtained small effect sizes suggest that these findings were significant only due the large sample size of the study. Therefore, these significant quantitative results should be interpreted with caution.

The second, third, and fourth research questions asked about the selected 15 middle school students’ emotional states (a) when Wuhan was locked down for the COVID-19 pandemic, (b) when the lockdown was lifted in Wuhan, and (c) the changes of their emotional states from the lockdown to the lifted lockdown, and then to today, i.e., 1-year after the lockdown was lifted. These 15 participants represented Grades 7, 8, and 9 student groups. Both similarities and differences were found among the three groups.

When Wuhan was locked down on January 23, 2020, all 15 participants except for one male student in Grade 7 group experienced worry, fear, feelings of loss of control, and negative emotions. The lockdown brought lifestyle changes to these middle school students. Home quarantine, reduced activities, online classes, and increased time with family members would create feelings of loss of control and negative emotions (Jiang et al., 2020; Wang et al., 2020).

On hearing the news that the lockdown was lifted on April 8, 2020, they felt cheerful, calm, relaxed, and excited. However, they became further anxious and worried because they thought they might experience the lockdown again. Unlike his peers, the male student in Grade 7 group felt sad about it because his parents had to go out to work and he would hardly see them at home.

After the lockdown was lifted, schools resumed and their life went back to normal. Their feelings changed over the past year, from being surprised, fearful, terrified schools when Wuhan was locked down, to being happy, cheerful, and excited when the lockdown was lifted, and to being worried, relaxed, and proud 1 year after the lifted lockdown. Grades 8 and 9 students are feeling relaxed and proud today because the pandemic is back in control and living in China becomes safe. However, several Grade 7 students explained that they are still feeling worried even today because the pandemic is still ongoing globally and it has become a serious threat to their mental health.

The present study was limited in the following three ways. First, it examined Wuhan middle school students’ emotional states during the COVID-19 pandemic only from a student perspective. Their parents’ perceptions were not included in the study. Therefore, the results should be interpreted with caution. Second, due to the limited availability and accessibility of participants, the second phase of data collection (i.e., focus group interviews) involved only 15 middle school students; this small sample size may limit the generalization of the findings to all the middle school students in Wuhan. Third, the interviews in this study were retrospective. Many factors may have distorted the participants’ answers in one way or the other when they reported about their emotional states in the past. Again, the results should be interpreted with caution.

In light of these limitations, the following two conclusions were reached. First, the middle school students in Wuhan experienced emotional changes during the COVID-19 pandemic when the city was locked down and they were home quarantined. During this major public health emergency, feelings of loss of control and negative emotions were commonly found in these students (Liu, 2020; Wang et al., 2020; Liu et al., 2021).

Second, the emotional states of middle school students in Wuhan changed substantially at different time nodes during this pandemic. They felt fearful and scared when the city was locked down; and these feelings were developed into feelings of loss of control and negative emotions (Liu Y. Y. et al., 2020; Main et al., 2011). They became happy and excited when the lockdown was lifted. During the 1 year after the lockdown was lifted, they were feeling relaxed and safe.

The results of this study have the important implications for the students, their parents, and the schools they attend. The middle school students should be aware of the threat of the major public health emergencies and be psychologically prepared for dealing with such emergencies. Their parents should notice their emotional changes during these emergencies and provide them with protection and guidance so that they can get over them successfully. The schools should also require their teachers and psychologists to provide students with guidance and consulting services so that they become psychologically and emotionally prepared before these emergencies occur.

## Data Availability Statement

The datasets presented in this article are not readily available because data belong to the funder. Requests to access the datasets should be directed to YH, 316678481@qq.com.

## Ethics Statement

Ethical review and approval was not required for the study on human participants in accordance with the local legislation and institutional requirements. Written informed consent to participate in this study was provided by the participants’ legal guardian/next of kin.

## Author Contributions

YH: study design, data collection, and research funding provider. JH: data analysis, drafting, editing, revising, and proofreading the entire manuscript. TW: literature search, drafting literature review section, and data collection. SX: data collection and data preparation. JL: partial data collection and data preparation. All authors contributed to the article and approved the submitted version.

## Conflict of Interest

The authors declare that the research was conducted in the absence of any commercial or financial relationships that could be construed as a potential conflict of interest.

## Publisher’s Note

All claims expressed in this article are solely those of the authors and do not necessarily represent those of their affiliated organizations, or those of the publisher, the editors and the reviewers. Any product that may be evaluated in this article, or claim that may be made by its manufacturer, is not guaranteed or endorsed by the publisher.

## Appendix

**TABLE A1:** Profiles of 22 public schools involved in the study.

District	School	Grade	# of students in school	# of teachers in school
Wuhan District A	School A	Grades 7–9	1,500	103
	School B	Grades 7–9	1,500	97
	School C	Grades 7–9	1,600	107
	School D	Grades 7–9	1,300	79
	School E	Grades 7–9	1,500	89
	School F	Grades 7–9	1,400	81
Wuhan District B	School A	Grades 7–9	1,300	71
	School B	Grades 7–9	1,300	76
	School C	Grades 7–9	1,400	83
	School D	Grades 7–9	1,500	101
	School E	Grades 7–9	1,600	109
	School F	Grades 7–9	1,400	98
Wuhan District C	School A	Grades 7–9	1,600	97
	School B	Grades 7–9	1,500	90
	School C	Grades 7–9	1,400	83
	School D	Grades 7–9	1,700	102
	School E	Grades 7–9	1,300	78
	School F	Grades 7–9	1,300	71
Wuhan District D	School A	Grades 7–9	1,700	112
	School B	Grades 7–9	1,500	98
	School C	Grades 7–9	1,500	92
	School D	Grades 7–9	1,400	89
